# Supplementary material for: Detailed molecular and epigenetic characterization of the pig IPEC-J2 and chicken SL-29 cell lines
Source: iScience. 2023 Feb 20;26(3):106252. doi: 10.1016/j.isci.2023.106252 (PMC10018572; doi:10.1016/j.isci.2023.106252)
Supplement: Data S2. Complete homer output for identified motifs in Chicken SL-29, related to Tables 5 and 6 — Homer motif analysis results for histone modifications H3K4me1, H3K4me3, H3K27ac, enhancers, and ATAC-seq of chicken SL-29 cell line. Parameters for possible false positives is as mentioned earlier for S5. [file mmc3.zip › Data_S2/S6/Chicken_SL_29/peak_files_ATAC/knownResults.html]

peak\_files - Homer Known Motif Enrichment Results


# Homer Known Motif Enrichment Results (peak\_files)

Homer *de novo* Motif Results  
Gene Ontology Enrichment Results  
Known Motif Enrichment Results (txt file)  
Total Target Sequences = 86973, Total Background Sequences = 86710

|  |  |  |  |  |  |  |  |  |  |  |  |
| --- | --- | --- | --- | --- | --- | --- | --- | --- | --- | --- | --- |
| Rank | Motif | Name | P-value | log P-pvalue | q-value (Benjamini) | # Target Sequences with Motif | % of Targets Sequences with Motif | # Background Sequences with Motif | % of Background Sequences with Motif | Motif File | SVG |
| 1 | T G C A A C T G T A C G A T G C A G T C G A C T T C G A A T C G | ZNF711(Zf)/SHSY5Y-ZNF711-ChIP-Seq(GSE20673)/Homer | 1e-176 | -4.063e+02 | 0.0000 | 24735.0 | 28.44% | 21018.4 | 24.24% | motif file (matrix) | svg |
| 2 | C T G A A C T G A C T G A G T C A G T C A G C T C T A G T A C G | ZFX(Zf)/mES-Zfx-ChIP-Seq(GSE11431)/Homer | 1e-153 | -3.541e+02 | 0.0000 | 17046.0 | 19.60% | 14050.7 | 16.21% | motif file (matrix) | svg |
| 3 | T C G A A C G T A C T G C T G A A G T C T C A G A G C T G T A C C G T A A G C T G A T C T C G A | JunD(bZIP)/K562-JunD-ChIP-Seq/Homer | 1e-148 | -3.420e+02 | 0.0000 | 1931.0 | 2.22% | 1004.5 | 1.16% | motif file (matrix) | svg |
| 4 | A T G C T C A G T C G A G C A T A C T G C G T A A G T C T C A G G A C T T G A C C G T A A G C T | Atf2(bZIP)/3T3L1-Atf2-ChIP-Seq(GSE56872)/Homer | 1e-125 | -2.883e+02 | 0.0000 | 5009.0 | 5.76% | 3525.7 | 4.07% | motif file (matrix) | svg |
| 5 | T G C A T C G A T C G A A C G T C A T G C G T A A G T C T C A G A C G T G T A C C G T A A G C T | CREB5(bZIP)/LNCaP-CREB5.V5-ChIP-Seq(GSE137775)/Homer | 1e-112 | -2.591e+02 | 0.0000 | 4794.0 | 5.51% | 3412.7 | 3.94% | motif file (matrix) | svg |
| 6 | T A C G T C G A G A C T A C T G C T G A A G T C T C A G G A C T T G A C C T G A | Atf1(bZIP)/K562-ATF1-ChIP-Seq(GSE31477)/Homer | 1e-109 | -2.516e+02 | 0.0000 | 8965.0 | 10.31% | 7078.3 | 8.16% | motif file (matrix) | svg |
| 7 | T C G A C T A G A G T C A G T C C G T A C G T A A C G T T A G C T C A G T A C G | NFY(CCAAT)/Promoter/Homer | 1e-108 | -2.506e+02 | 0.0000 | 10219.0 | 11.75% | 8210.2 | 9.47% | motif file (matrix) | svg |
| 8 | T C G A G C A T A C T G C T G A A G T C T C A G G A C T G T A C C G T A A G C T A G T C G A T C | c-Jun-CRE(bZIP)/K562-cJun-ChIP-Seq(GSE31477)/Homer | 1e-105 | -2.420e+02 | 0.0000 | 4555.0 | 5.24% | 3251.4 | 3.75% | motif file (matrix) | svg |
| 9 | A T G C A G C T T C A G T G A C T C A G A T G C T G C A A C G T A T C G G A T C A C T G A G T C | NRF1(NRF)/MCF7-NRF1-ChIP-Seq(Unpublished)/Homer | 1e-94 | -2.173e+02 | 0.0000 | 2331.0 | 2.68% | 1473.3 | 1.70% | motif file (matrix) | svg |
| 10 | T G C A A G C T C T G A A T C G G A C T C T A G G T A C G A T C G T C A A G T C G T A C G A C T C T A G A T C G G C A T C A T G C A T G G A T C G T A C C T G A | CTCF(Zf)/CD4+-CTCF-ChIP-Seq(Barski\_et\_al.)/Homer | 1e-93 | -2.155e+02 | 0.0000 | 2517.0 | 2.89% | 1624.6 | 1.87% | motif file (matrix) | svg |
| 11 | A T C G A G C T A C T G A G T C A C T G A G T C C G T A A C G T A C T G A G T C A C T G A G T C | NRF(NRF)/Promoter/Homer | 1e-90 | -2.084e+02 | 0.0000 | 2771.0 | 3.19% | 1842.2 | 2.12% | motif file (matrix) | svg |
| 12 | T A G C C T A G T C G A G A C T A C T G C T G A A G T C T C A G G C A T T G A C C T G A A G C T | Atf7(bZIP)/3T3L1-Atf7-ChIP-Seq(GSE56872)/Homer | 1e-85 | -1.961e+02 | 0.0000 | 6426.0 | 7.39% | 5006.8 | 5.77% | motif file (matrix) | svg |
| 13 | T A G C A G T C T G A C A G T C C T A G A T C G A G T C C A T G T G A C A G T C G T A C A G T C A G T C G C A T C T A G A T C G G C A T A C T G A T C G G A T C | BORIS(Zf)/K562-CTCFL-ChIP-Seq(GSE32465)/Homer | 1e-81 | -1.872e+02 | 0.0000 | 4187.0 | 4.81% | 3077.5 | 3.55% | motif file (matrix) | svg |
| 14 | A G T C G A C T C A G T A C T G C T A G T G A C G C T A A T G C G C A T A T C G C G A T A C T G G A T C G T A C G T C A C T G A | NF1(CTF)/LNCAP-NF1-ChIP-Seq(Unpublished)/Homer | 1e-64 | -1.484e+02 | 0.0000 | 6323.0 | 7.27% | 5086.1 | 5.87% | motif file (matrix) | svg |
| 15 | T A G C C G T A C T G A T A C G C G T A A C G T A C T G A C T G A G T C T A C G C T A G G T A C | YY1(Zf)/Promoter/Homer | 1e-63 | -1.471e+02 | 0.0000 | 1440.0 | 1.66% | 889.5 | 1.03% | motif file (matrix) | svg |
| 16 | T C G A C A T G C A T G A C G T A T G C T C G A C T G A A G C T T A C G T G C A G T A C G A T C A G C T A G T C | FXR(NR),IR1/Liver-FXR-ChIP-Seq(Chong\_et\_al.)/Homer | 1e-61 | -1.405e+02 | 0.0000 | 6369.0 | 7.32% | 5159.7 | 5.95% | motif file (matrix) | svg |
| 17 | T C A G C T A G C T A G A C T G A C T G G A T C A C T G A C T G C T A G C T A G A G T C G A T C | KLF1(Zf)/HUDEP2-KLF1-CutnRun(GSE136251)/Homer | 1e-59 | -1.369e+02 | 0.0000 | 12086.0 | 13.90% | 10452.5 | 12.06% | motif file (matrix) | svg |
| 18 | G A C T A G T C C G A T A C T G C T G A T G A C G T A C C G T A A T C G G C A T C T G A C T A G | Bcl11a(Zf)/HSPC-BCL11A-ChIP-Seq(GSE104676)/Homer | 1e-58 | -1.342e+02 | 0.0000 | 9652.0 | 11.10% | 8197.0 | 9.45% | motif file (matrix) | svg |
| 19 | C T G A T G C A T A G C T G A C T A C G T C A G C T G A G C T A T C A G G A C T | ELF1(ETS)/Jurkat-ELF1-ChIP-Seq(SRA014231)/Homer | 1e-52 | -1.203e+02 | 0.0000 | 5693.0 | 6.55% | 4631.6 | 5.34% | motif file (matrix) | svg |
| 20 | A C T G C A T G G C T A T C G A G C T A A G C T A G C T G T A C A G T C T G A C | NFkB-p65-Rel(RHD)/ThioMac-LPS-Expression(GSE23622)/Homer | 1e-48 | -1.128e+02 | 0.0000 | 898.0 | 1.03% | 525.0 | 0.61% | motif file (matrix) | svg |
| 21 | G C T A C G A T C A T G A T G C A G T C A G T C G A C T T A C G T C G A C T A G A C T G T A G C | AP-2alpha(AP2)/Hela-AP2alpha-ChIP-Seq(GSE31477)/Homer | 1e-46 | -1.079e+02 | 0.0000 | 14784.0 | 17.00% | 13188.0 | 15.21% | motif file (matrix) | svg |
| 22 | T C A G C G T A A G T C A G C T C G T A A G T C C T G A C G T A A G T C G C A T A G T C A G T C A G T C C T G A A C T G T G C A T C G A C A T G A T C G G A T C | Ronin(THAP)/ES-Thap11-ChIP-Seq(GSE51522)/Homer | 1e-45 | -1.051e+02 | 0.0000 | 521.0 | 0.60% | 259.9 | 0.30% | motif file (matrix) | svg |
| 23 | G C A T C T A G G T A C A G T C C G A T A C T G C T A G C T A G G T A C G C T A | ZNF416(Zf)/HEK293-ZNF416.GFP-ChIP-Seq(GSE58341)/Homer | 1e-44 | -1.018e+02 | 0.0000 | 22223.0 | 25.55% | 20387.5 | 23.51% | motif file (matrix) | svg |
| 24 | G A T C T C G A A G T C C G A T C G A T A G T C A T G C A C T G A T C G G A C T | Elk1(ETS)/Hela-Elk1-ChIP-Seq(GSE31477)/Homer | 1e-40 | -9.233e+01 | 0.0000 | 5808.0 | 6.68% | 4862.3 | 5.61% | motif file (matrix) | svg |
| 25 | T G C A T C G A T A G C G T A C T C A G C T A G G T C A G C T A T C A G G A C T | ETS(ETS)/Promoter/Homer | 1e-39 | -9.072e+01 | 0.0000 | 3007.0 | 3.46% | 2341.6 | 2.70% | motif file (matrix) | svg |
| 26 | A T C G A G T C A G T C G A C T A T G C C T G A C T A G A C T G T A C G G T A C C T G A C G A T | AP-2gamma(AP2)/MCF7-TFAP2C-ChIP-Seq(GSE21234)/Homer | 1e-36 | -8.339e+01 | 0.0000 | 18579.0 | 21.36% | 17027.7 | 19.64% | motif file (matrix) | svg |
| 27 | T C G A C G T A A G T C A G C T C G T A A G T C T C G A G C T A G A C T C G A T A G T C A G T C A G T C C T G A T C A G T G C A T C G A C A G T A T C G A G T C | GFY-Staf(?,Zf)/Promoter/Homer | 1e-34 | -8.006e+01 | 0.0000 | 698.0 | 0.80% | 419.4 | 0.48% | motif file (matrix) | svg |
| 28 | T A C G T A C G G T A C A T C G T A C G T A C G G T C A C T G A C G T A G A C T | E2F4(E2F)/K562-E2F4-ChIP-Seq(GSE31477)/Homer | 1e-34 | -7.964e+01 | 0.0000 | 6058.0 | 6.96% | 5158.1 | 5.95% | motif file (matrix) | svg |
| 29 | A G T C C T G A A G T C C G A T C A G T G A T C A T G C A C T G A T C G G A C T | Fli1(ETS)/CD8-FLI-ChIP-Seq(GSE20898)/Homer | 1e-34 | -7.886e+01 | 0.0000 | 12440.0 | 14.30% | 11173.0 | 12.89% | motif file (matrix) | svg |
| 30 | G A T C C T G A A G T C C G A T C G A T G A T C A G T C A C T G A T C G A G C T | Elk4(ETS)/Hela-Elk4-ChIP-Seq(GSE31477)/Homer | 1e-34 | -7.857e+01 | 0.0000 | 5699.0 | 6.55% | 4832.1 | 5.57% | motif file (matrix) | svg |
| 31 | C G A T C T A G C T G A A T G C C T G A T C G A C G T A C T G A T C G A T A G C A G T C C G T A A C T G T C G A A T G C | Hand2(bHLH)/Mesoderm-Hand2-ChIP-Seq(GSE61475)/Homer | 1e-33 | -7.761e+01 | 0.0000 | 6920.0 | 7.96% | 5970.2 | 6.89% | motif file (matrix) | svg |
| 32 | A T G C A T C G T A C G A G C T A T C G C T G A A G T C C T A G A G C T A T G C C T G A A T G C | CRE(bZIP)/Promoter/Homer | 1e-33 | -7.622e+01 | 0.0000 | 3616.0 | 4.16% | 2940.4 | 3.39% | motif file (matrix) | svg |
| 33 | C T A G T A C G A G T C C G T A A G T C A C G T A G T C T C G A C G T A T A C G | Nkx2.1(Homeobox)/LungAC-Nkx2.1-ChIP-Seq(GSE43252)/Homer | 1e-32 | -7.369e+01 | 0.0000 | 33645.0 | 38.68% | 31851.9 | 36.74% | motif file (matrix) | svg |
| 34 | A T G C A G T C G C T A C G A T C G T A G C A T G C T A C G A T C T A G C A T G T G A C G T C A | CArG(MADS)/PUER-Srf-ChIP-Seq(Sullivan\_et\_al.)/Homer | 1e-31 | -7.349e+01 | 0.0000 | 3219.0 | 3.70% | 2594.8 | 2.99% | motif file (matrix) | svg |
| 35 | C G T A C T G A A C T G A G C T A G T C G A T C G A T C G C A T C T G A C T A G C T A G T A C G T C G A T G C A G C A T | EBF2(EBF)/BrownAdipose-EBF2-ChIP-Seq(GSE97114)/Homer | 1e-31 | -7.338e+01 | 0.0000 | 13825.0 | 15.89% | 12541.0 | 14.46% | motif file (matrix) | svg |
| 36 | T C G A T A G C T G C A A C T G A C T G C G T A C G T A C T A G G A C T T A C G | ETS1(ETS)/Jurkat-ETS1-ChIP-Seq(GSE17954)/Homer | 1e-31 | -7.318e+01 | 0.0000 | 12675.0 | 14.57% | 11442.4 | 13.20% | motif file (matrix) | svg |
| 37 | A G T C C T A G C T A G A G T C G A T C G T A C A G T C C T A G A G T C A G T C A G T C G T A C | Sp2(Zf)/HEK293-Sp2.eGFP-ChIP-Seq(Encode)/Homer | 1e-29 | -6.887e+01 | 0.0000 | 22810.0 | 26.23% | 21278.0 | 24.54% | motif file (matrix) | svg |
| 38 | C G T A C T A G A C T G A C T G G A C T C T A G C A G T C T A G C A T G G A T C | KLF5(Zf)/LoVo-KLF5-ChIP-Seq(GSE49402)/Homer | 1e-27 | -6.222e+01 | 0.0000 | 17100.0 | 19.66% | 15801.7 | 18.23% | motif file (matrix) | svg |
| 39 | A T C G T C G A G A C T A T C G T G A C A C G T C T A G A C T G C G T A A C T G A G T C G T A C | ZNF415(Zf)/HEK293-ZNF415.GFP-ChIP-Seq(GSE58341)/Homer | 1e-26 | -6.202e+01 | 0.0000 | 10782.0 | 12.40% | 9727.6 | 11.22% | motif file (matrix) | svg |
| 40 | C T A G T C G A A C G T A C T G C G T A T A G C C G A T G T A C C G T A A G C T G A T C G T A C | Jun-AP1(bZIP)/K562-cJun-ChIP-Seq(GSE31477)/Homer | 1e-25 | -5.929e+01 | 0.0000 | 2200.0 | 2.53% | 1739.0 | 2.01% | motif file (matrix) | svg |
| 41 | T A G C C G A T A C G T A G C T A G C T A G T C A T G C A G T C A C T G A T G C A T G C G C T A | E2F7(E2F)/Hela-E2F7-ChIP-Seq(GSE32673)/Homer | 1e-25 | -5.926e+01 | 0.0000 | 1606.0 | 1.85% | 1217.5 | 1.40% | motif file (matrix) | svg |
| 42 | C T A G T C G A C G A T A C T G C G T A T A C G A G C T T G A C G C T A A C G T G A T C T A G C | Fosl2(bZIP)/3T3L1-Fosl2-ChIP-Seq(GSE56872)/Homer | 1e-25 | -5.922e+01 | 0.0000 | 3063.0 | 3.52% | 2514.6 | 2.90% | motif file (matrix) | svg |
| 43 | T A G C C G A T T A C G A C T G A G T C A C T G A T C G A T C G C G T A C T G A | E2F1(E2F)/Hela-E2F1-ChIP-Seq(GSE22478)/Homer | 1e-25 | -5.851e+01 | 0.0000 | 3590.0 | 4.13% | 2997.3 | 3.46% | motif file (matrix) | svg |
| 44 | A T G C G A T C G A C T A G C T C G A T C G A T G T C A C G A T T C G A A T C G T A G C T A G C | TATA-Box(TBP)/Promoter/Homer | 1e-25 | -5.830e+01 | 0.0000 | 11700.0 | 13.45% | 10637.9 | 12.27% | motif file (matrix) | svg |
| 45 | G T A C C A G T A C T G A C T G A C T G G A T C A C T G A C G T A C T G A C T G A G T C G A T C | KLF6(Zf)/PDAC-KLF6-ChIP-Seq(GSE64557)/Homer | 1e-25 | -5.769e+01 | 0.0000 | 16138.0 | 18.55% | 14917.1 | 17.21% | motif file (matrix) | svg |
| 46 | A G T C T G C A T C G A C T G A A C T G C A T G A C G T A T G C G T C A T A C G | Erra(NR)/HepG2-Erra-ChIP-Seq(GSE31477)/Homer | 1e-25 | -5.766e+01 | 0.0000 | 27173.0 | 31.24% | 25679.2 | 29.62% | motif file (matrix) | svg |
| 47 | T G A C C G T A C T G A A C T G A C T G G A C T G A T C T G C A G T A C T A C G | SF1(NR)/H295R-Nr5a1-ChIP-Seq(GSE44220)/Homer | 1e-24 | -5.728e+01 | 0.0000 | 7248.0 | 8.33% | 6410.7 | 7.39% | motif file (matrix) | svg |
| 48 | T A C G C T A G A T G C G A T C G T A C A G T C C T A G A G T C A G T C A G T C G T A C A G T C | Sp1(Zf)/Promoter/Homer | 1e-24 | -5.709e+01 | 0.0000 | 5619.0 | 6.46% | 4882.7 | 5.63% | motif file (matrix) | svg |
| 49 | T C A G A G C T A T G C C G T A A G C T T C A G C A G T A C T G C T G A A G T C | MITF(bHLH)/MastCells-MITF-ChIP-Seq(GSE48085)/Homer | 1e-24 | -5.649e+01 | 0.0000 | 10431.0 | 11.99% | 9439.6 | 10.89% | motif file (matrix) | svg |
| 50 | T C G A G A C T A G C T T G A C A G C T G T A C T C A G G A T C A T C G T G C A A C T G C T G A | GFX(?)/Promoter/Homer | 1e-23 | -5.501e+01 | 0.0000 | 241.0 | 0.28% | 115.1 | 0.13% | motif file (matrix) | svg |
| 51 | C G T A T A C G T C G A A C T G A C T G C G T A C G T A T A C G A G C T T A C G | PU.1(ETS)/ThioMac-PU.1-ChIP-Seq(GSE21512)/Homer | 1e-22 | -5.286e+01 | 0.0000 | 5913.0 | 6.80% | 5185.4 | 5.98% | motif file (matrix) | svg |
| 52 | C T A G A G C T G A C T C A T G A G T C A G T C G T C A C A G T C T A G T C A G G T A C C T G A T C G A G A T C T G A C | Rfx2(HTH)/LoVo-RFX2-ChIP-Seq(GSE49402)/Homer | 1e-22 | -5.071e+01 | 0.0000 | 1397.0 | 1.61% | 1062.2 | 1.23% | motif file (matrix) | svg |
| 53 | T A C G T C G A C A G T A C T G G C T A A T G C C G A T G T A C C G T A A C T G T A G C C G T A | NF-E2(bZIP)/K562-NFE2-ChIP-Seq(GSE31477)/Homer | 1e-21 | -4.918e+01 | 0.0000 | 831.0 | 0.96% | 583.5 | 0.67% | motif file (matrix) | svg |
| 54 | A G T C A T C G C T A G A G C T G A C T C T A G A G T C A G T C G C T A C A G T T C A G T C A G G A T C C T G A T C G A G A T C | RFX(HTH)/K562-RFX3-ChIP-Seq(SRA012198)/Homer | 1e-20 | -4.739e+01 | 0.0000 | 1240.0 | 1.43% | 936.9 | 1.08% | motif file (matrix) | svg |
| 55 | T C G A A C G T C A T G G C T A T A G C C G A T G T A C G C T A A C G T A T G C | AP-1(bZIP)/ThioMac-PU.1-ChIP-Seq(GSE21512)/Homer | 1e-20 | -4.732e+01 | 0.0000 | 6914.0 | 7.95% | 6169.1 | 7.12% | motif file (matrix) | svg |
| 56 | T C A G A C G T A G T C T C G A A G T C T C A G G C A T C T A G C T A G A G C T | Usf2(bHLH)/C2C12-Usf2-ChIP-Seq(GSE36030)/Homer | 1e-20 | -4.682e+01 | 0.0000 | 3765.0 | 4.33% | 3220.6 | 3.71% | motif file (matrix) | svg |
| 57 | T C G A T C G A T A G C G T A C T C A G T A C G C G T A C G T A T C A G A G C T | GABPA(ETS)/Jurkat-GABPa-ChIP-Seq(GSE17954)/Homer | 1e-19 | -4.406e+01 | 0.0000 | 9645.0 | 11.09% | 8801.0 | 10.15% | motif file (matrix) | svg |
| 58 | T A C G T A C G G T A C A T C G A C T G T A C G T C G A C T G A T C G A A T C G | E2F6(E2F)/Hela-E2F6-ChIP-Seq(GSE31477)/Homer | 1e-19 | -4.391e+01 | 0.0000 | 8151.0 | 9.37% | 7373.5 | 8.50% | motif file (matrix) | svg |
| 59 | C G T A C T G A T A C G G A C T G A T C T C G A G A T C G A T C T G C A G A C T T C G A C G T A A T C G A G T C C G A T C G T A C G T A G T C A C G T A C T A G | PSE(SNAPc)/K562-mStart-Seq/Homer | 1e-18 | -4.343e+01 | 0.0000 | 4791.0 | 5.51% | 4197.3 | 4.84% | motif file (matrix) | svg |
| 60 | T C A G G C T A T C A G C A G T T G A C G T C A A G T C A T C G T G C A G T A C C A G T G A T C | Npas4(bHLH)/Neuron-Npas4-ChIP-Seq(GSE127793)/Homer | 1e-18 | -4.243e+01 | 0.0000 | 12709.0 | 14.61% | 11766.7 | 13.57% | motif file (matrix) | svg |
| 61 | G C T A C G T A A G T C A C G T T C G A T A C G A C T G A G C T A G T C T C G A | RORgt(NR)/EL4-RORgt.Flag-ChIP-Seq(GSE56019)/Homer | 1e-17 | -4.064e+01 | 0.0000 | 1438.0 | 1.65% | 1132.3 | 1.31% | motif file (matrix) | svg |
| 62 | G C T A C G T A A G T C A C G T T C G A T A C G A C T G A G C T A G T C T C G A | RORgt(NR)/EL4-RORgt.Flag-ChIP-Seq(GSE56019)/Homer | 1e-17 | -4.064e+01 | 0.0000 | 1438.0 | 1.65% | 1132.3 | 1.31% | motif file (matrix) | svg |
| 63 | A G C T A G C T C A T G C T G A G T A C A G T C A G C T A G C T C A G T C T A G | RARa(NR)/K562-RARa-ChIP-Seq(Encode)/Homer | 1e-17 | -4.048e+01 | 0.0000 | 28809.0 | 33.12% | 27529.6 | 31.75% | motif file (matrix) | svg |
| 64 | G C T A A G T C T A C G T G C A A T C G T C A G G C T A T C G A T C A G A G C T | ELF5(ETS)/T47D-ELF5-ChIP-Seq(GSE30407)/Homer | 1e-17 | -4.037e+01 | 0.0000 | 7973.0 | 9.17% | 7235.8 | 8.35% | motif file (matrix) | svg |
| 65 | T C A G T G A C G T A C T G C A G T A C C T A G G T A C A T G C A G T C G T C A A G T C G A C T | Klf9(Zf)/GBM-Klf9-ChIP-Seq(GSE62211)/Homer | 1e-17 | -3.925e+01 | 0.0000 | 4563.0 | 5.25% | 4012.1 | 4.63% | motif file (matrix) | svg |
| 66 | T C A G A G C T A T G C C G T A A G T C T C A G A C G T A T C G T C G A A G T C G A T C T G A C | TFE3(bHLH)/MEF-TFE3-ChIP-Seq(GSE75757)/Homer | 1e-16 | -3.866e+01 | 0.0000 | 735.0 | 0.85% | 528.7 | 0.61% | motif file (matrix) | svg |
| 67 | C A T G C T A G T C G A A C G T A C T G C G T A T A G C C G A T T G A C C G T A A G C T G A T C | Fra2(bZIP)/Striatum-Fra2-ChIP-Seq(GSE43429)/Homer | 1e-16 | -3.858e+01 | 0.0000 | 4143.0 | 4.76% | 3623.7 | 4.18% | motif file (matrix) | svg |
| 68 | A C T G C T A G T C G A C G A T C A T G G C T A A T C G C G A T G T A C G C T A A G C T G T A C | Fra1(bZIP)/BT549-Fra1-ChIP-Seq(GSE46166)/Homer | 1e-15 | -3.652e+01 | 0.0000 | 4607.0 | 5.30% | 4074.0 | 4.70% | motif file (matrix) | svg |
| 69 | G A C T T C A G C T A G A G T C A G T C G T A C A G T C C T G A A G T C A G T C A G T C G A C T A G T C A C T G A T G C | KLF3(Zf)/MEF-Klf3-ChIP-Seq(GSE44748)/Homer | 1e-15 | -3.578e+01 | 0.0000 | 6411.0 | 7.37% | 5787.7 | 6.68% | motif file (matrix) | svg |
| 70 | G A C T T C G A C G A T T A C G C T A G T A C G A C T G A T G C G T A C G T A C | Zac1(Zf)/Neuro2A-Plagl1-ChIP-Seq(GSE75942)/Homer | 1e-15 | -3.545e+01 | 0.0000 | 37870.0 | 43.54% | 36578.4 | 42.19% | motif file (matrix) | svg |
| 71 | T C A G G T C A G C T A A G T C C G T A A T C G T C G A T C A G C G T A A C T G A C T G A C T G | ZNF768(Zf)/Rajj-ZNF768-ChIP-Seq(GSE111879)/Homer | 1e-15 | -3.471e+01 | 0.0000 | 1157.0 | 1.33% | 905.2 | 1.04% | motif file (matrix) | svg |
| 72 | C G T A C A T G C A T G A C T G C T A G T C G A G C A T C G A T A G C T A G T C G A T C G T A C | NFkB-p65(RHD)/GM12787-p65-ChIP-Seq(GSE19485)/Homer | 1e-14 | -3.362e+01 | 0.0000 | 6591.0 | 7.58% | 5978.0 | 6.89% | motif file (matrix) | svg |
| 73 | A G C T A G T C A G T C A C G T C T A G A C G T A C G T A C G T C G T A A G T C G A T C C G T A | FOXP1(Forkhead)/H9-FOXP1-ChIP-Seq(GSE31006)/Homer | 1e-14 | -3.338e+01 | 0.0000 | 3463.0 | 3.98% | 3022.6 | 3.49% | motif file (matrix) | svg |
| 74 | C T G A T A G C T G A C T C A G C T A G G T C A C G T A T C A G A G C T T C A G | ETV4(ETS)/HepG2-ETV4-ChIP-Seq(ENCODE)/Homer | 1e-14 | -3.331e+01 | 0.0000 | 12937.0 | 14.87% | 12095.7 | 13.95% | motif file (matrix) | svg |
| 75 | T C G A T G C A A C T G G T C A A G T C A G T C A G T C A G T C A G C T T G A C | LRF(Zf)/Erythroblasts-ZBTB7A-ChIP-Seq(GSE74977)/Homer | 1e-14 | -3.290e+01 | 0.0000 | 21672.0 | 24.92% | 20629.4 | 23.79% | motif file (matrix) | svg |
| 76 | A G C T A G C T T A G C A T C G A G T C A C T G A T G C A T C G T C G A C T G A T C G A C T G A | E2F(E2F)/Hela-CellCycle-Expression/Homer | 1e-13 | -3.194e+01 | 0.0000 | 491.0 | 0.56% | 340.3 | 0.39% | motif file (matrix) | svg |
| 77 | A G T C C G A T A C T G A T C G T G A C G C T A C A T G A T C G T G A C C G A T A C T G T A G C G T A C G T C A | Tlx?(NR)/NPC-H3K4me1-ChIP-Seq(GSE16256)/Homer | 1e-13 | -3.125e+01 | 0.0000 | 6353.0 | 7.30% | 5773.2 | 6.66% | motif file (matrix) | svg |
| 78 | C T A G T C G A G C A T C A T G G C T A T A G C C G A T G T A C C T G A A G C T | JunB(bZIP)/DendriticCells-Junb-ChIP-Seq(GSE36099)/Homer | 1e-13 | -3.098e+01 | 0.0000 | 4544.0 | 5.22% | 4056.3 | 4.68% | motif file (matrix) | svg |
| 79 | A C G T G A C T T A G C C G T A C T G A C A T G C T A G G A C T G A T C C G T A | Nr5a2(NR)/Pancreas-LRH1-ChIP-Seq(GSE34295)/Homer | 1e-13 | -3.020e+01 | 0.0000 | 8758.0 | 10.07% | 8091.5 | 9.33% | motif file (matrix) | svg |
| 80 | A C T G A C G T C A T G A T C G A T C G T G A C A C T G A T C G A T C G T G C A C T G A C G T A | E2F3(E2F)/MEF-E2F3-ChIP-Seq(GSE71376)/Homer | 1e-13 | -3.001e+01 | 0.0000 | 9095.0 | 10.46% | 8418.1 | 9.71% | motif file (matrix) | svg |
| 81 | C A G T T G C A A C G T A C T G C G T A A T C G C G A T T G A C C G T A A C G T | BATF(bZIP)/Th17-BATF-ChIP-Seq(GSE39756)/Homer | 1e-12 | -2.863e+01 | 0.0000 | 5204.0 | 5.98% | 4702.4 | 5.42% | motif file (matrix) | svg |
| 82 | C G T A C G T A C G T A G C A T G C A T T A C G G T A C G A C T A C T G C G T A A T C G A C G T G T A C C G T A A G C T | Bach1(bZIP)/K562-Bach1-ChIP-Seq(GSE31477)/Homer | 1e-11 | -2.755e+01 | 0.0000 | 691.0 | 0.79% | 521.7 | 0.60% | motif file (matrix) | svg |
| 83 | G T C A G C A T A C T G G T A C G A C T A C T G G C T A A T C G C A G T G T A C C G T A A G C T | Nrf2(bZIP)/Lymphoblast-Nrf2-ChIP-Seq(GSE37589)/Homer | 1e-11 | -2.734e+01 | 0.0000 | 623.0 | 0.72% | 463.7 | 0.53% | motif file (matrix) | svg |
| 84 | T C G A T A G C G T C A A C T G C T A G C G T A C G A T A C T G A C G T A C T G A C T G A C G T | ETS:RUNX(ETS,Runt)/Jurkat-RUNX1-ChIP-Seq(GSE17954)/Homer | 1e-11 | -2.665e+01 | 0.0000 | 1328.0 | 1.53% | 1091.0 | 1.26% | motif file (matrix) | svg |
| 85 | A C T G C A T G T C G A A C G T A C T G C G T A A T C G A C G T G T A C C G T A G A C T A G T C | Fos(bZIP)/TSC-Fos-ChIP-Seq(GSE110950)/Homer | 1e-11 | -2.608e+01 | 0.0000 | 4875.0 | 5.60% | 4412.3 | 5.09% | motif file (matrix) | svg |
| 86 | A T G C A G T C C T G A A G T C C G A T A C G T A G T C A G T C A C G T A T C G G A C T A C G T | Etv2(ETS)/ES-ER71-ChIP-Seq(GSE59402)/Homer | 1e-10 | -2.477e+01 | 0.0000 | 10027.0 | 11.53% | 9384.2 | 10.82% | motif file (matrix) | svg |
| 87 | T A C G T C A G T G C A A G C T T G A C A G C T A G T C A C T G G A T C A C T G T C G A A C T G C T G A C T G A A T G C | ZBTB33(Zf)/GM12878-ZBTB33-ChIP-Seq(GSE32465)/Homer | 1e-10 | -2.470e+01 | 0.0000 | 542.0 | 0.62% | 401.2 | 0.46% | motif file (matrix) | svg |
| 88 | A C G T G A C T A T G C G C T A C T G A C T A G A C T G G A C T A G T C C G T A | Nr5a2(NR)/mES-Nr5a2-ChIP-Seq(GSE19019)/Homer | 1e-10 | -2.451e+01 | 0.0000 | 6353.0 | 7.30% | 5841.3 | 6.74% | motif file (matrix) | svg |
| 89 | C G T A C G T A C G T A G C A T G C A T A C T G G T A C G A C T C T A G G C T A T A C G G A C T T G A C C G T A A G C T | NFE2L2(bZIP)/HepG2-NFE2L2-ChIP-Seq(Encode)/Homer | 1e-10 | -2.374e+01 | 0.0000 | 606.0 | 0.70% | 459.9 | 0.53% | motif file (matrix) | svg |
| 90 | C T A G T C G A A C G T A C T G C G T A A T G C A C G T G T A C C G T A A G C T G A T C G T A C | Atf3(bZIP)/GBM-ATF3-ChIP-Seq(GSE33912)/Homer | 1e-10 | -2.352e+01 | 0.0000 | 5490.0 | 6.31% | 5024.4 | 5.80% | motif file (matrix) | svg |
| 91 | C G A T C T A G A C G T G T C A C G T A C G T A A G T C C G T A | Foxo3(Forkhead)/U2OS-Foxo3-ChIP-Seq(E-MTAB-2701)/Homer | 1e-9 | -2.250e+01 | 0.0000 | 5603.0 | 6.44% | 5143.8 | 5.93% | motif file (matrix) | svg |
| 92 | A T G C A T G C A T C G T A C G A G C T A G T C G C T A A G T C T C A G G A C T A C T G T C G A | E-box(bHLH)/Promoter/Homer | 1e-9 | -2.224e+01 | 0.0000 | 1086.0 | 1.25% | 891.6 | 1.03% | motif file (matrix) | svg |
| 93 | C T G A T C A G C T G A C T A G C A T G A C G T A T G C C G T A A T G C G C A T T C A G C T G A A C T G A C G T C A G T A G T C C G T A C A G T C T A G C A T G | VDR(NR),DR3/GM10855-VDR+vitD-ChIP-Seq(GSE22484)/Homer | 1e-9 | -2.147e+01 | 0.0000 | 3948.0 | 4.54% | 3572.6 | 4.12% | motif file (matrix) | svg |
| 94 | T C G A A C G T A C G T C T G A G A T C T C A G G A C T G T C A C G T A A G C T G T C A C T A G A G C T A C G T T C G A | NFIL3(bZIP)/HepG2-NFIL3-ChIP-Seq(Encode)/Homer | 1e-9 | -2.110e+01 | 0.0000 | 4974.0 | 5.72% | 4555.9 | 5.25% | motif file (matrix) | svg |
| 95 | T G C A C T A G C T A G C T G A C A T G A C T G T G C A G A T C G T C A T G C A G T C A G T C A A G C T C T A G G C A T | ZNF675(Zf)/HEK293-ZNF675.GFP-ChIP-Seq(GSE58341)/Homer | 1e-9 | -2.103e+01 | 0.0000 | 1683.0 | 1.93% | 1444.9 | 1.67% | motif file (matrix) | svg |
| 96 | T A C G G A C T A C T G A C T G C T A G A T G C A G T C A G T C A G T C C T G A | ZNF692(Zf)/HEK293-ZNF692.GFP-ChIP-Seq(GSE58341)/Homer | 1e-8 | -2.063e+01 | 0.0000 | 2014.0 | 2.32% | 1754.1 | 2.02% | motif file (matrix) | svg |
| 97 | T A C G A T G C G A C T A C T G A G C T A G T C G T C A T G C A A C G T A G T C G C T A T G C A | Pknox1(Homeobox)/ES-Prep1-ChIP-Seq(GSE63282)/Homer | 1e-8 | -1.949e+01 | 0.0000 | 2826.0 | 3.25% | 2525.7 | 2.91% | motif file (matrix) | svg |
| 98 | T C A G C A T G C A T G A C T G A C T G A G C T A C T G A C G T A C T G C A G T A T G C A G T C | KLF10(Zf)/HEK293-KLF10.GFP-ChIP-Seq(GSE58341)/Homer | 1e-8 | -1.939e+01 | 0.0000 | 6352.0 | 7.30% | 5900.0 | 6.80% | motif file (matrix) | svg |
| 99 | C G A T T A C G T G A C G A C T C A T G C G T A T A C G A C G T G T A C C T G A | Bach2(bZIP)/OCILy7-Bach2-ChIP-Seq(GSE44420)/Homer | 1e-8 | -1.894e+01 | 0.0000 | 1959.0 | 2.25% | 1714.6 | 1.98% | motif file (matrix) | svg |
| 100 | G A T C G C T A C A G T A C G T T A C G A G T C A T G C C T A G A G T C T C G A | Zfp57(Zf)/H1-ZFP57.HA-ChIP-Seq(GSE115387)/Homer | 1e-7 | -1.821e+01 | 0.0000 | 7930.0 | 9.12% | 7441.6 | 8.58% | motif file (matrix) | svg |
| 101 | T G C A T A G C G A C T T G C A T G A C T G C A C G T A A G C T A G C T A G T C A G T C G T A C | GFY(?)/Promoter/Homer | 1e-7 | -1.789e+01 | 0.0000 | 841.0 | 0.97% | 689.6 | 0.80% | motif file (matrix) | svg |
| 102 | T C G A T A G C G T C A A C T G A C T G C G T A C G T A C T A G A G C T T C A G | ERG(ETS)/VCaP-ERG-ChIP-Seq(GSE14097)/Homer | 1e-7 | -1.786e+01 | 0.0000 | 17971.0 | 20.66% | 17263.5 | 19.91% | motif file (matrix) | svg |
| 103 | G A C T C T A G G A T C C A G T A C T G C T G A A T G C G C A T A T G C C T G A | MafA(bZIP)/Islet-MafA-ChIP-Seq(GSE30298)/Homer | 1e-7 | -1.644e+01 | 0.0000 | 13659.0 | 15.70% | 13059.4 | 15.06% | motif file (matrix) | svg |
| 104 | T G C A C T G A C A T G C T A G C A G T A G T C C G T A A T G C A T G C T A C G G C A T T C A G G T C A G A T C G T A C | ERE(NR),IR3/MCF7-ERa-ChIP-Seq(Unpublished)/Homer | 1e-7 | -1.642e+01 | 0.0000 | 4161.0 | 4.78% | 3827.0 | 4.41% | motif file (matrix) | svg |
| 105 | G T A C G C T A T C A G C T G A C T A G C A T G A G C T G A T C T G C A T C G A C T G A A C T G C A G T A G T C G A T C G C T A | HNF4a(NR),DR1/HepG2-HNF4a-ChIP-Seq(GSE25021)/Homer | 1e-7 | -1.624e+01 | 0.0000 | 4209.0 | 4.84% | 3874.1 | 4.47% | motif file (matrix) | svg |
| 106 | G A T C G C A T A C T G C T A G C T G A A G C T G C T A G C T A C T G A T C A G G C A T T G C A A C G T A C G T G A T C G A C T G C A T C T A G T A C G G A C T C T A G C A T G C T A G G T A C T C G A | ZNF136(Zf)/HEK293-ZNF136.GFP-ChIP-Seq(GSE58341)/Homer | 1e-7 | -1.622e+01 | 0.0000 | 870.0 | 1.00% | 723.1 | 0.83% | motif file (matrix) | svg |
| 107 | T C G A C T G A T A G C T G A C T C A G T C A G C G T A C G T A T C A G A G C T | ETV1(ETS)/GIST48-ETV1-ChIP-Seq(GSE22441)/Homer | 1e-6 | -1.578e+01 | 0.0000 | 14794.0 | 17.01% | 14185.0 | 16.36% | motif file (matrix) | svg |
| 108 | C T G A T C A G C A G T C T A G A C T G C T A G G A T C A T C G A C T G C T G A T C A G G A T C | Sp5(Zf)/mES-Sp5.Flag-ChIP-Seq(GSE72989)/Homer | 1e-6 | -1.513e+01 | 0.0000 | 14062.0 | 16.17% | 13481.0 | 15.55% | motif file (matrix) | svg |
| 109 | T G A C C G A T A C T G A C T G A C T G G A C T A C T G A C G T A C T G A C T G G A T C G A T C | EKLF(Zf)/Erythrocyte-Klf1-ChIP-Seq(GSE20478)/Homer | 1e-6 | -1.513e+01 | 0.0000 | 1640.0 | 1.89% | 1442.3 | 1.66% | motif file (matrix) | svg |
| 110 | A C T G T C A G A G C T G A C T C A T G A G T C A G T C G C T A C G A T C T A G T C A G G T A C C T G A T C G A | Rfx1(HTH)/NPC-H3K4me1-ChIP-Seq(GSE16256)/Homer | 1e-6 | -1.505e+01 | 0.0000 | 2722.0 | 3.13% | 2465.7 | 2.84% | motif file (matrix) | svg |
| 111 | T C G A A C T G C A T G A G C T A G T C C G T A C T G A C T A G A C T G C G A T A T G C C T G A | RAR:RXR(NR),DR0/ES-RAR-ChIP-Seq(GSE56893)/Homer | 1e-5 | -1.316e+01 | 0.0000 | 1132.0 | 1.30% | 981.1 | 1.13% | motif file (matrix) | svg |
| 112 | T A C G A C T G A G C T G T A C C G T A T C G A C T G A A C T G C A T G A C G T A G T C C G T A | COUP-TFII(NR)/K562-NR2F1-ChIP-Seq(Encode)/Homer | 1e-5 | -1.314e+01 | 0.0000 | 16989.0 | 19.53% | 16401.1 | 18.92% | motif file (matrix) | svg |
| 113 | G C A T A T C G C A T G G T A C G C T A A G T C T C A G T G A C G T C A T G C A | Arnt:Ahr(bHLH)/MCF7-Arnt-ChIP-Seq(Lo\_et\_al.)/Homer | 1e-5 | -1.226e+01 | 0.0000 | 10454.0 | 12.02% | 10002.2 | 11.54% | motif file (matrix) | svg |
| 114 | T G C A A G C T A C G T C T A G G A T C C T A G G A T C G T C A C T G A A G T C | CEBP(bZIP)/ThioMac-CEBPb-ChIP-Seq(GSE21512)/Homer | 1e-5 | -1.191e+01 | 0.0000 | 5951.0 | 6.84% | 5614.5 | 6.48% | motif file (matrix) | svg |
| 115 | C T G A A G T C C G A T A G C T A T G C G T A C A C G T A T C G C A G T G C A T | Elf4(ETS)/BMDM-Elf4-ChIP-Seq(GSE88699)/Homer | 1e-5 | -1.189e+01 | 0.0000 | 10184.0 | 11.71% | 9745.3 | 11.24% | motif file (matrix) | svg |
| 116 | G A T C C G T A G A C T C T A G G A T C C T G A G A C T C T G A G A C T C T A G G A T C C T G A G A C T C T G A G A C T | OCT:OCT(POU,Homeobox)/NPC-OCT6-ChIP-Seq(GSE43916)/Homer | 1e-5 | -1.186e+01 | 0.0000 | 507.0 | 0.58% | 414.2 | 0.48% | motif file (matrix) | svg |
| 117 | C T G A C A G T C T G A A G T C C T A G G A C T A T C G G T A C | HIF-1b(HLH)/T47D-HIF1b-ChIP-Seq(GSE59937)/Homer | 1e-4 | -1.144e+01 | 0.0000 | 18130.0 | 20.84% | 17568.7 | 20.26% | motif file (matrix) | svg |
| 118 | A T G C G A C T A G C T C T A G C G T A C T A G C G A T C T A G A T C G G A T C | Nkx2.2(Homeobox)/NPC-Nkx2.2-ChIP-Seq(GSE61673)/Homer | 1e-4 | -1.073e+01 | 0.0001 | 22916.0 | 26.35% | 22316.6 | 25.74% | motif file (matrix) | svg |
| 119 | C G T A T A G C T A G C T G C A A C T G C T A G C G T A C G T A T C A G G A C T | EHF(ETS)/LoVo-EHF-ChIP-Seq(GSE49402)/Homer | 1e-4 | -1.072e+01 | 0.0001 | 13425.0 | 15.43% | 12952.4 | 14.94% | motif file (matrix) | svg |
| 120 | C A T G C T A G A G C T G A C T C A T G A G T C G A T C G C T A C G A T C T A G T C A G G T A C C T G A T C G A | X-box(HTH)/NPC-H3K4me1-ChIP-Seq(GSE16256)/Homer | 1e-4 | -1.035e+01 | 0.0001 | 1182.0 | 1.36% | 1047.3 | 1.21% | motif file (matrix) | svg |
| 121 | C T G A C T G A T A G C G A T C G C T A G T A C A C G T G A T C T G C A C G T A | Nkx2.5(Homeobox)/HL1-Nkx2.5.biotin-ChIP-Seq(GSE21529)/Homer | 1e-4 | -1.011e+01 | 0.0001 | 24772.0 | 28.48% | 24172.1 | 27.88% | motif file (matrix) | svg |
| 122 | T A C G T G C A A G T C C G T A A C G T T G A C A C G T A C T G A C T G G C A T | TCF4(bHLH)/SHSY5Y-TCF4-ChIP-Seq(GSE96915)/Homer | 1e-4 | -9.951e+00 | 0.0002 | 23969.0 | 27.56% | 23382.6 | 26.97% | motif file (matrix) | svg |
| 123 | T C G A T C A G T C G A A C T G C A T G A C G T A G T C C T G A | COUP-TFII(NR)/Artia-Nr2f2-ChIP-Seq(GSE46497)/Homer | 1e-4 | -9.359e+00 | 0.0003 | 20510.0 | 23.58% | 19978.9 | 23.04% | motif file (matrix) | svg |
| 124 | T A C G T C A G A G C T A T G C C G T A A G T C T C A G A C G T A C T G T C G A | USF1(bHLH)/GM12878-Usf1-ChIP-Seq(GSE32465)/Homer | 1e-3 | -9.124e+00 | 0.0004 | 6289.0 | 7.23% | 5991.7 | 6.91% | motif file (matrix) | svg |
| 125 | A C G T T A C G G A T C A C T G A C G T C T A G A C T G A C T G G A T C C T A G C A T G C T A G | Egr2(Zf)/Thymocytes-Egr2-ChIP-Seq(GSE34254)/Homer | 1e-3 | -9.049e+00 | 0.0004 | 2378.0 | 2.73% | 2198.4 | 2.54% | motif file (matrix) | svg |
| 126 | T C G A T C G A T A C G G A T C G T C A G T A C C G A T A G C T G T C A T G C A | Nkx3.1(Homeobox)/LNCaP-Nkx3.1-ChIP-Seq(GSE28264)/Homer | 1e-3 | -8.668e+00 | 0.0006 | 26437.0 | 30.40% | 25870.0 | 29.84% | motif file (matrix) | svg |
| 127 | A G C T G A C T C T A G C G T A C A T G C G A T C T A G A T C G G A C T C A G T | Bapx1(Homeobox)/VertebralCol-Bapx1-ChIP-Seq(GSE36672)/Homer | 1e-3 | -8.244e+00 | 0.0009 | 22965.0 | 26.40% | 22444.7 | 25.89% | motif file (matrix) | svg |
| 128 | C T A G C T A G A G T C T C A G A C T G A C G T A C G T C T G A | MYB(HTH)/ERMYB-Myb-ChIPSeq(GSE22095)/Homer | 1e-3 | -7.668e+00 | 0.0016 | 21633.0 | 24.87% | 21145.2 | 24.39% | motif file (matrix) | svg |
| 129 | C A T G A C G T A G T C G A T C G A T C G A T C G C T A C T A G C T A G C T A G T C A G T C G A | EBF1(EBF)/Near-E2A-ChIP-Seq(GSE21512)/Homer | 1e-3 | -7.105e+00 | 0.0028 | 16606.0 | 19.09% | 16191.7 | 18.68% | motif file (matrix) | svg |
| 130 | T A G C G C A T A G T C G A T C A T G C G A C T C T A G A C T G A C T G C T G A A C T G C T A G A G T C T G A C C G A T | GLIS3(Zf)/Thyroid-Glis3.GFP-ChIP-Seq(GSE103297)/Homer | 1e-3 | -7.098e+00 | 0.0028 | 25458.0 | 29.27% | 24957.0 | 28.78% | motif file (matrix) | svg |
| 131 | G C A T G C A T C T G A A C G T C T G A A C G T C G T A C G T A C G T A A G T C G T C A G T C A | Foxf1(Forkhead)/Lung-Foxf1-ChIP-Seq(GSE77951)/Homer | 1e-2 | -6.804e+00 | 0.0037 | 6062.0 | 6.97% | 5816.1 | 6.71% | motif file (matrix) | svg |
| 132 | T C A G T C A G A C G T G T A C G C T A T C A G C T G A A C T G A C T G A G C T A G T C C G T A | EAR2(NR)/K562-NR2F6-ChIP-Seq(Encode)/Homer | 1e-2 | -6.619e+00 | 0.0044 | 15188.0 | 17.46% | 14806.1 | 17.08% | motif file (matrix) | svg |
| 133 | C T A G C T G A A G T C G C T A C G A T A C T G G A C T G A T C G A T C C T G A C T A G C T G A T G A C G C T A C G A T T C A G G A C T G A T C G A T C T G A C | p53(p53)/Saos-p53-ChIP-Seq(GSE15780)/Homer | 1e-2 | -6.599e+00 | 0.0045 | 1199.0 | 1.38% | 1095.6 | 1.26% | motif file (matrix) | svg |
| 134 | C T A G C T G A A G T C G C T A C G A T A C T G G A C T G A T C G A T C C T G A C T A G C T G A T G A C G C T A C G A T T C A G G A C T G A T C G A T C T G A C | p53(p53)/Saos-p53-ChIP-Seq/Homer | 1e-2 | -6.599e+00 | 0.0045 | 1199.0 | 1.38% | 1095.6 | 1.26% | motif file (matrix) | svg |
| 135 | C G T A C T G A C T A G C T G A A G T C G C T A C G A T A T C G G A C T G A T C A G T C C T G A C T A G C T A G A G T C G C T A C G A T C T A G G A T C G A T C | p73(p53)/Trachea-p73-ChIP-Seq(PRJNA310161)/Homer | 1e-2 | -6.554e+00 | 0.0046 | 734.0 | 0.84% | 654.1 | 0.75% | motif file (matrix) | svg |
| 136 | C T A G T C A G C A G T T C A G A C T G A C T G G A T C C T A G A C T G C T A G T C A G A T G C | KLF14(Zf)/HEK293-KLF14.GFP-ChIP-Seq(GSE58341)/Homer | 1e-2 | -6.497e+00 | 0.0049 | 24700.0 | 28.40% | 24229.6 | 27.95% | motif file (matrix) | svg |
| 137 | C T A G G A T C T C G A A T G C T C G A A G C T C T G A A C T G A C G T C T G A A C T G A C T G A C G T A G T C G A T C | ZKSCAN1(Zf)/HepG2-ZKSCAN1-ChIP-Seq(Encode)/Homer | 1e-2 | -6.464e+00 | 0.0050 | 242.0 | 0.28% | 198.2 | 0.23% | motif file (matrix) | svg |
| 138 | T A C G C T G A C A T G G A T C G T A C G C A T T C A G T A C G A G C T G T C A G A T C G C A T T A C G C G T A C T A G G A T C G A T C C G A T A C T G T C A G | ZNF322(Zf)/HEK293-ZNF322.GFP-ChIP-Seq(GSE58341)/Homer | 1e-2 | -6.450e+00 | 0.0050 | 4747.0 | 5.46% | 4537.0 | 5.23% | motif file (matrix) | svg |
| 139 | T C A G T A G C G A C T C A T G C T G A A T C G G C A T G T A C C G T A A C T G T A G C T G C A | MafK(bZIP)/C2C12-MafK-ChIP-Seq(GSE36030)/Homer | 1e-2 | -6.250e+00 | 0.0061 | 2416.0 | 2.78% | 2272.0 | 2.62% | motif file (matrix) | svg |
| 140 | T C A G C T A G C T A G C T A G T C A G T C G A C T G A C G A T A G T C G A T C A G T C T G A C | NFkB-p50,p52(RHD)/Monocyte-p50-ChIP-Chip(Schreiber\_et\_al.)/Homer | 1e-2 | -5.720e+00 | 0.0103 | 2249.0 | 2.59% | 2117.3 | 2.44% | motif file (matrix) | svg |
| 141 | C T G A C T G A C T A G T C G A C G T A A T G C C G T A A C T G C G T A A C G T C T G A C G A T A G C T C G T A A C G T A G T C C G A T T A C G G T C A G C A T | GATA(Zf),IR3/iTreg-Gata3-ChIP-Seq(GSE20898)/Homer | 1e-2 | -5.189e+00 | 0.0174 | 1302.0 | 1.50% | 1209.4 | 1.39% | motif file (matrix) | svg |
| 142 | C T G A T A G C G C T A C G A T A T C G A G T C G A T C G A T C C T A G T C A G T C A G G T A C G C T A C A G T | p53(p53)/mES-cMyc-ChIP-Seq(GSE11431)/Homer | 1e-2 | -5.181e+00 | 0.0174 | 276.0 | 0.32% | 235.6 | 0.27% | motif file (matrix) | svg |
| 143 | C G T A A C T G C G T A A C G T A T C G C A G T T A G C C G T A T C G A G T A C C T G A T A G C C G T A A C T G C G T A A C G T C G T A C T G A A T C G G C T A | GATA3(Zf),DR8/iTreg-Gata3-ChIP-Seq(GSE20898)/Homer | 1e-2 | -5.168e+00 | 0.0175 | 783.0 | 0.90% | 713.0 | 0.82% | motif file (matrix) | svg |
| 144 | T G A C C T G A C T A G T C G A C T G A A T G C C G T A A C T G G C A T G T A C G C A T A T C G G C A T A G C T G A T C | PR(NR)/T47D-PR-ChIP-Seq(GSE31130)/Homer | 1e-2 | -4.966e+00 | 0.0213 | 28535.0 | 32.81% | 28105.0 | 32.42% | motif file (matrix) | svg |
| 145 | G T C A T C G A C T A G C T A G A G T C G T C A C G A T C T A G G A C T G A T C G A T C T C A G C T A G C T G A A G T C G C T A C A G T T C A G G A T C G A T C | p63(p53)/Keratinocyte-p63-ChIP-Seq(GSE17611)/Homer | 1e-2 | -4.868e+00 | 0.0233 | 4363.0 | 5.02% | 4195.7 | 4.84% | motif file (matrix) | svg |
| 146 | A G C T C T G A C T A G C T A G A C T G T A G C T G C A T C G A C T G A C T A G C A T G A C G T A T G C T C G A | RXR(NR),DR1/3T3L1-RXR-ChIP-Seq(GSE13511)/Homer | 1e-2 | -4.779e+00 | 0.0253 | 14191.0 | 16.32% | 13887.7 | 16.02% | motif file (matrix) | svg |
